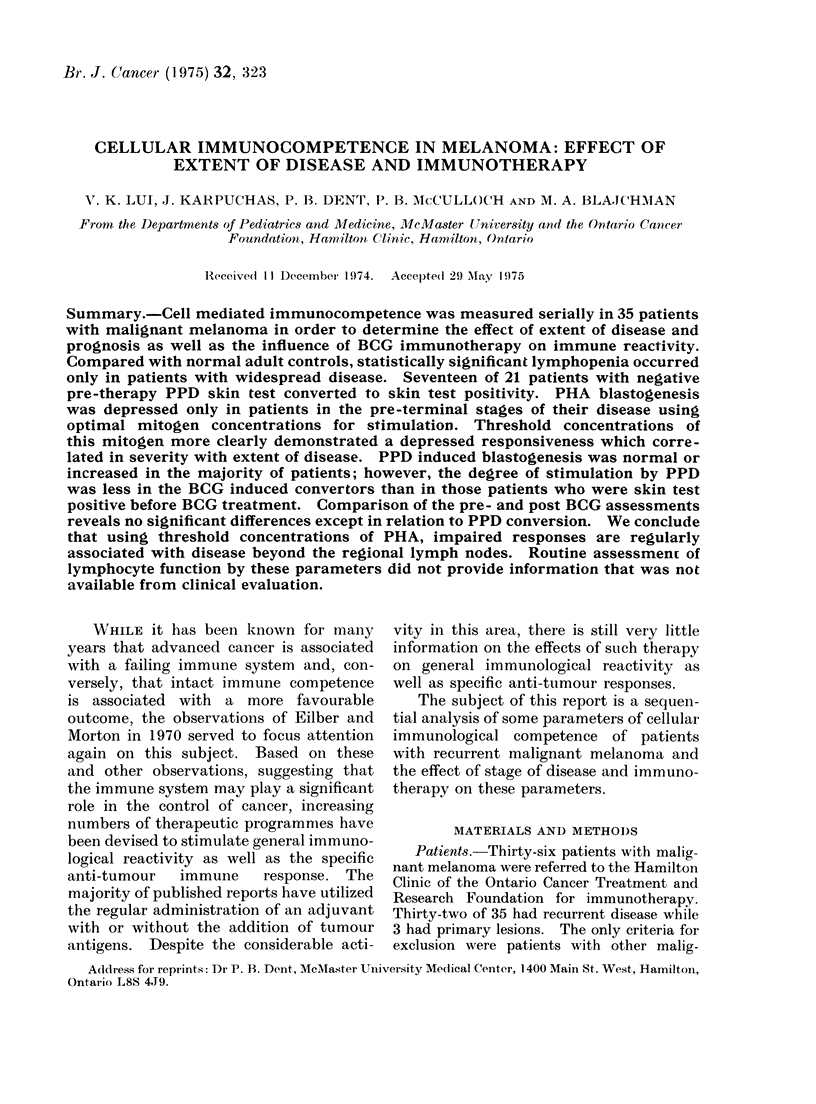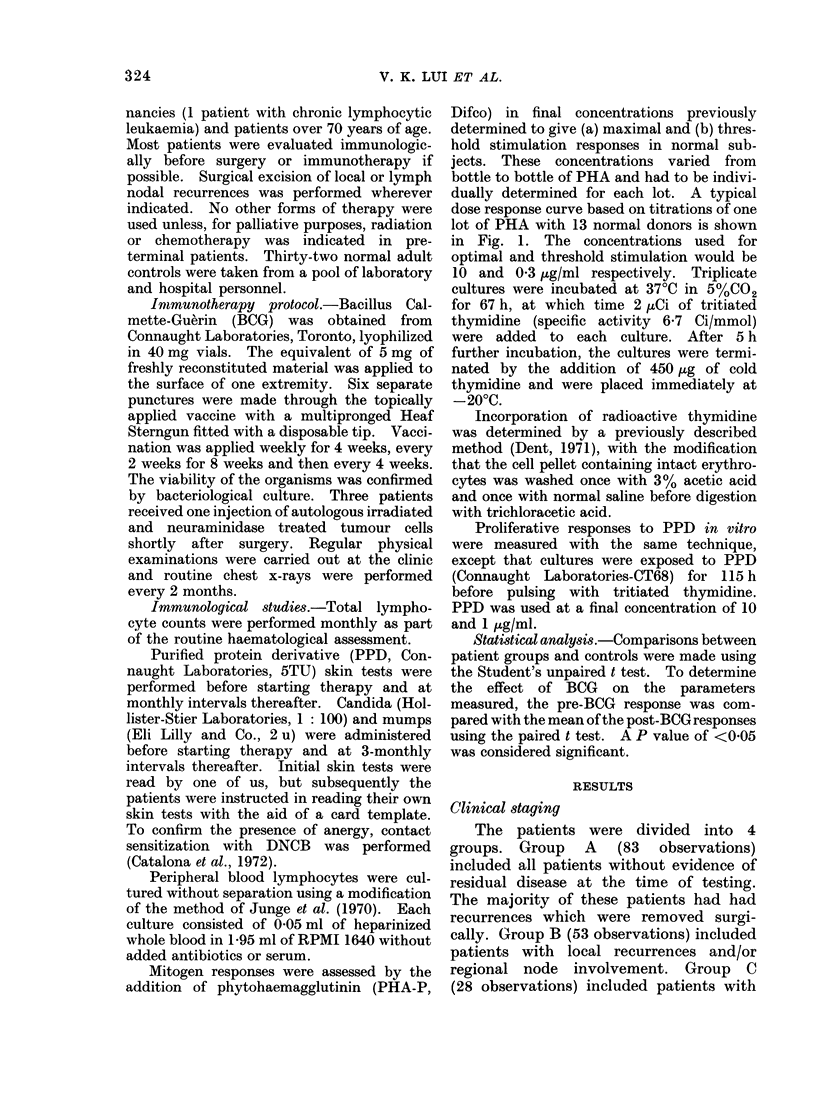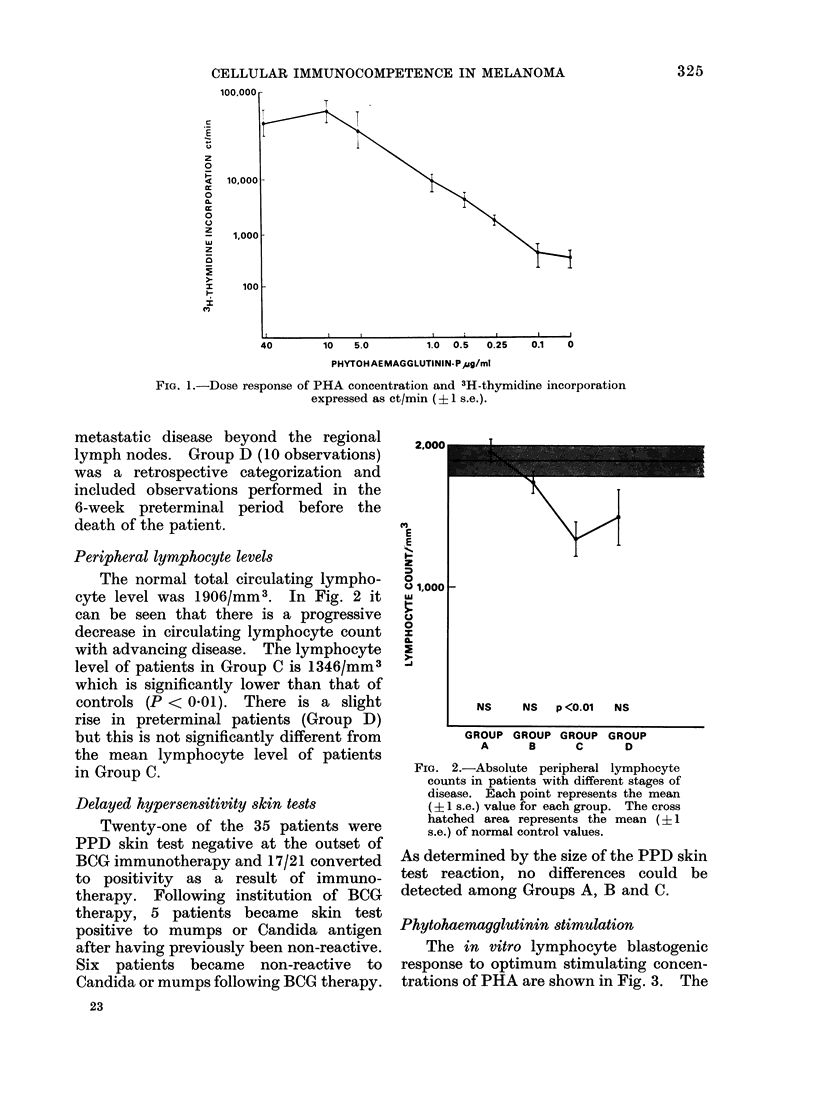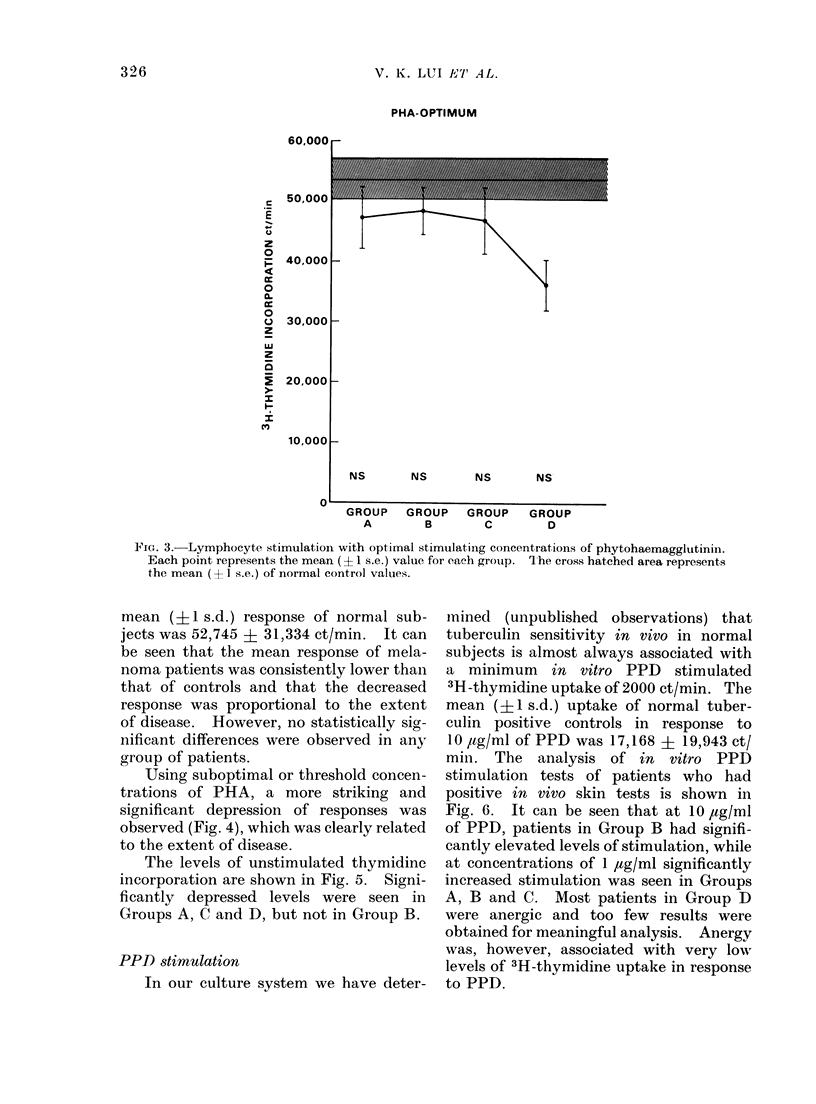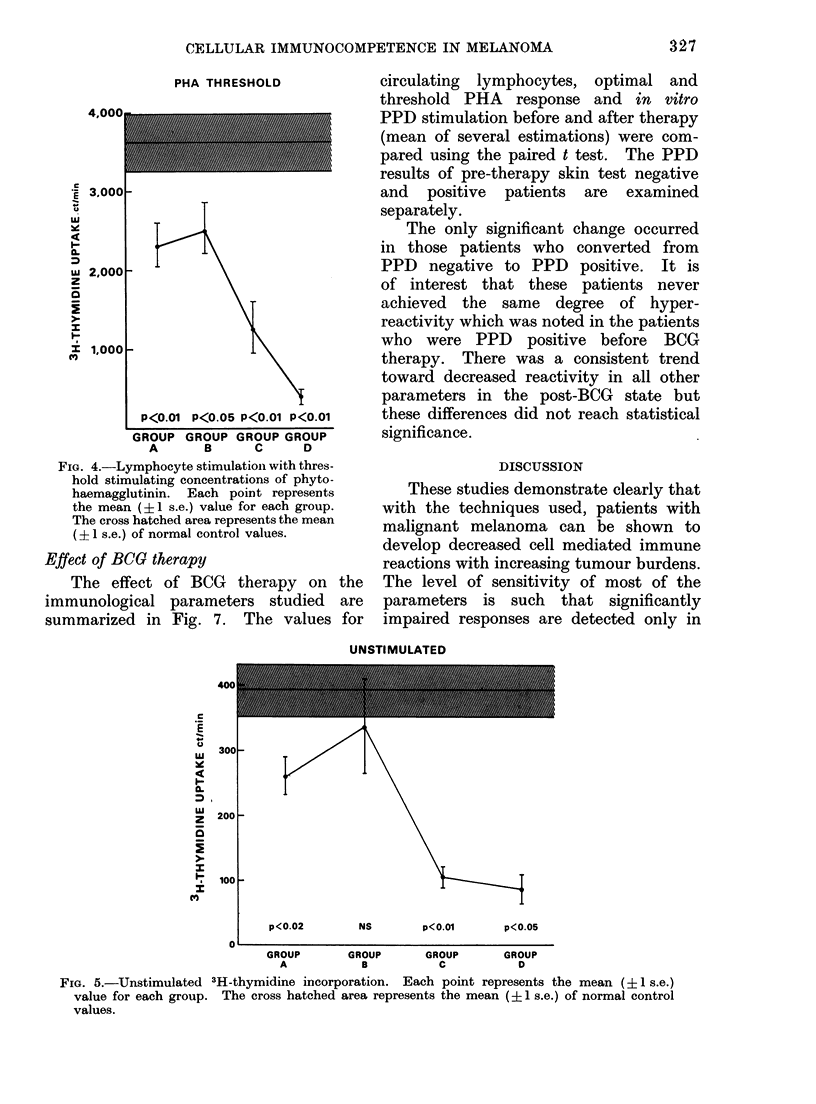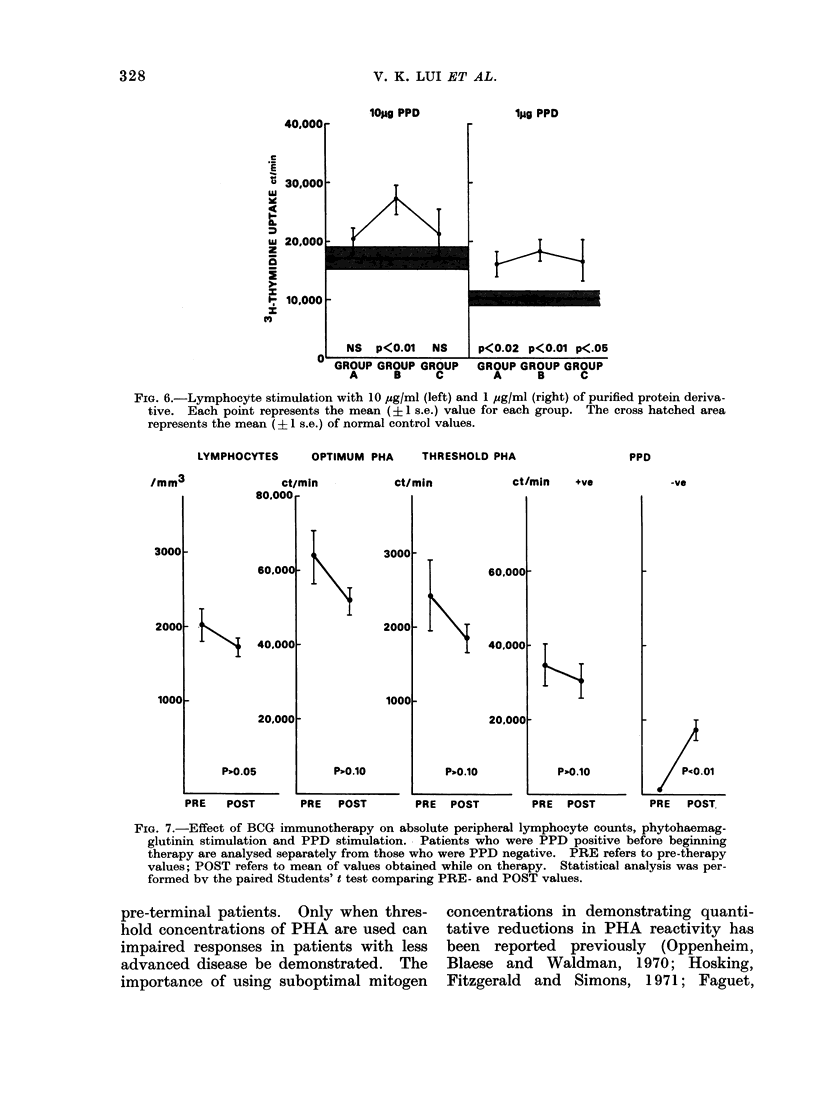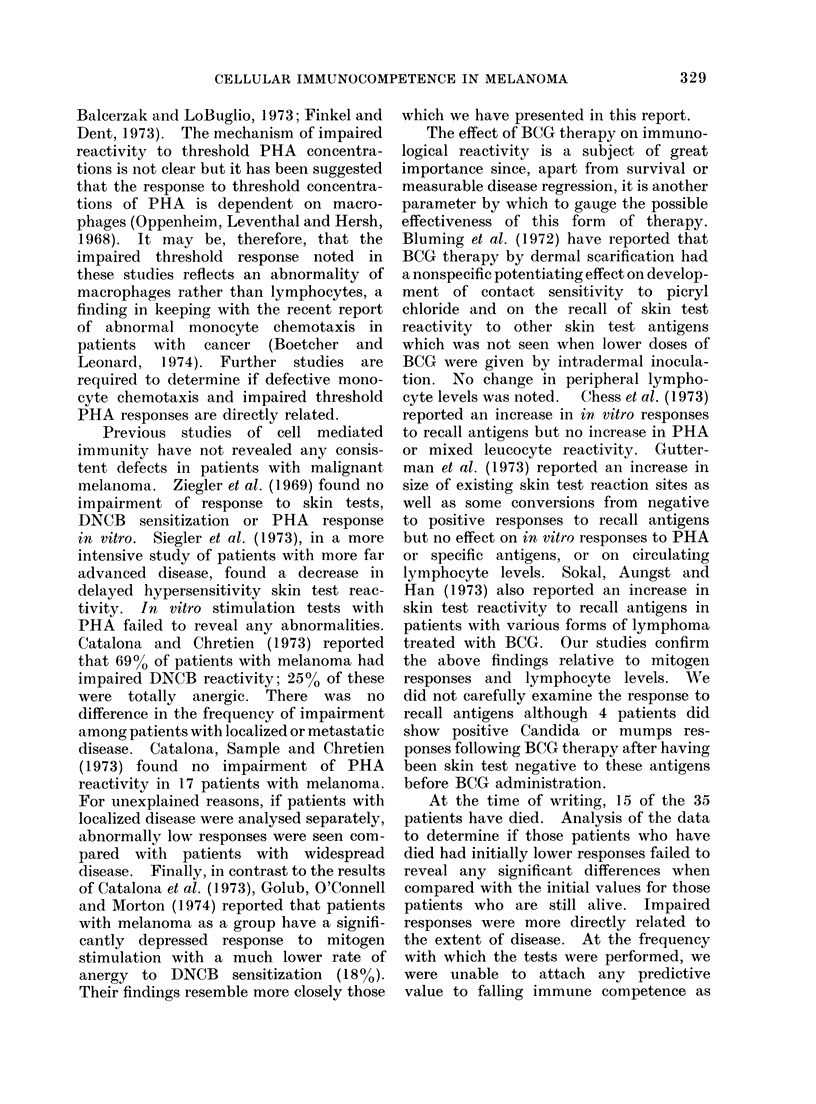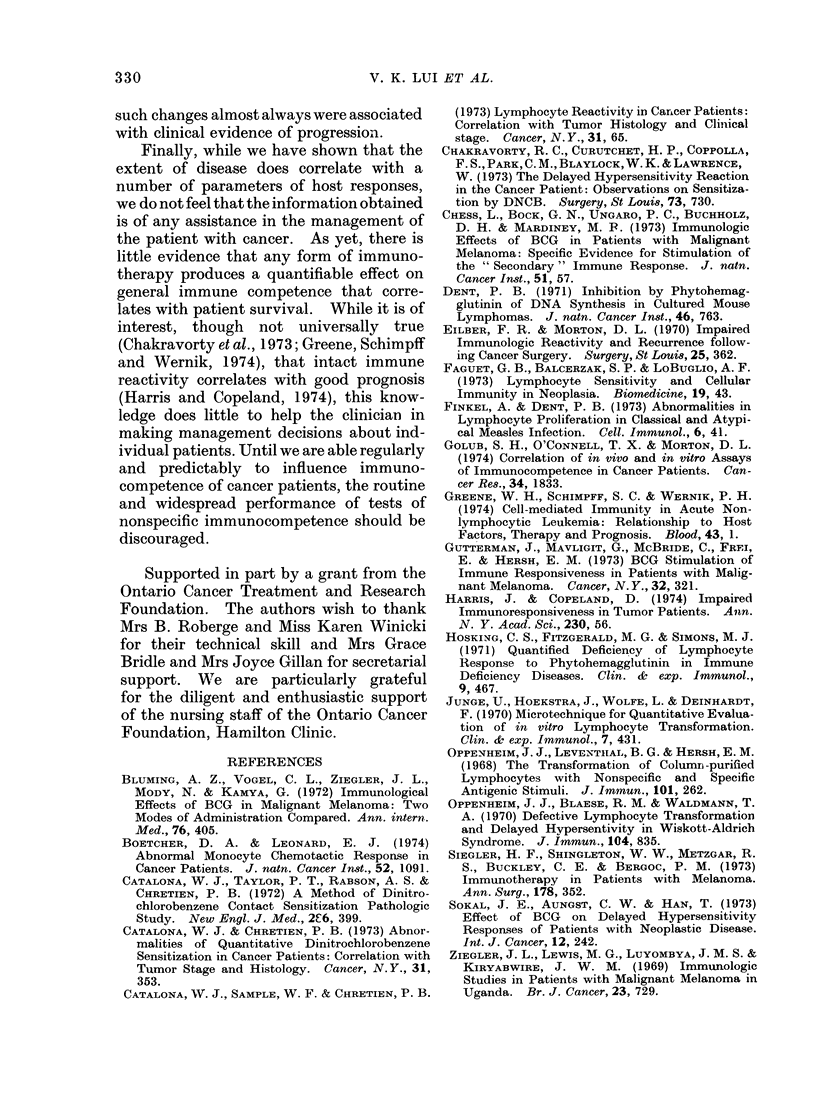# Cellular immunocompetence in melanoma: effect of extent of disease and immunotherapy.

**DOI:** 10.1038/bjc.1975.230

**Published:** 1975-09

**Authors:** V. K. Lui, J. Karpuchas, P. B. Dent, P. B. McCulloch, M. A. Blajchman

## Abstract

Cell mediated immunocompetence was measured serially in 35 patients with malignant melanoma in order to determine the effect of extent of disease and prognosis as well as the influence of BCG immunotherapy on immune reactivity. Compared with normal adult controls, statistically significant lymphopenia occurred only in patients with widespread disease. Seventeen of 21 patients with negative pre-therapy PPD skin test converted to skin test positivity. PHA blastogenesis was depressed only in patients in the pre-terminal stages of their disease using optimal mitogen concentrations for stimulation. Threshold concentrations of this mitogen more clearly demonstrated a depressed responsiveness which correlated in severity with extent of disease. PPD induced blastogenesis was normal or increased in the majority of patients; however, the degree of stimulation by PPD was less in the BCG induced convertors than in those patients who were skin test positive before BCG treatment. Comparison of the pre- and post BCG assessments reveals no significant differences except in relation to PPD conversion. We conclude that using threshold concentrations of PHA, impaired responses are regularly associated with disease beyond the regional lymph nodes. Routine assessment of lymphocyte function by these parameters did not provide information that was not available from clinical evaluation.


*Br. J. Cancer* (1975) **32**, 323

### CELLULAR IMMUNOCOMPETENCE IN MELANOMA: EFFECT OF EXTENT OF DISEASE AND IMMUNOTHERAPY

V. K. LUI, J. KARPUCHAS, P. B. DENT, P. B. McCULLOCH AND M. A. BLAJCHMAN

*From the Departments of Pediatrics and Medicine, McMaster University and the Ontario Cancer Foundation, Hamilton Clinic, Hamilton, Ontario*

Received 11 December 1974. Accepted 29 May 1975

**Summary.—Cell mediated immunocompetence was measured serially in 35 patients with malignant melanoma in order to determine the effect of extent of disease and prognosis as well as the influence of BCG immunotherapy on immune reactivity. Compared with normal adult controls, statistically significant lymphopenia occurred only in patients with widespread disease. Seventeen of 21 patients with negative pre-therapy PPD skin test converted to skin test positivity. PHA blastogenesis was depressed only in patients in the pre-terminal stages of their disease using optimal mitogen concentrations for stimulation. Threshold concentrations of this mitogen more clearly demonstrated a depressed responsiveness which correlated in severity with extent of disease. PPD induced blastogenesis was normal or increased in the majority of patients; however, the degree of stimulation by PPD was less in the BCG induced convertors than in those patients who were skin test positive before BCG treatment. Comparison of the pre- and post BCG assessments reveals no significant differences except in relation to PPD conversion. We conclude that using threshold concentrations of PHA, impaired responses are regularly associated with disease beyond the regional lymph nodes. Routine assessment of lymphocyte function by these parameters did not provide information that was not available from clinical evaluation.**

WHILE it has been known for many years that advanced cancer is associated with a failing immune system and, conversely, that intact immune competence is associated with a more favourable outcome, the observations of Eilber and Morton in 1970 served to focus attention again on this subject. Based on these and other observations, suggesting that the immune system may play a significant role in the control of cancer, increasing numbers of therapeutic programmes have been devised to stimulate general immunological reactivity as well as the specific anti-tumour immune response. The majority of published reports have utilized the regular administration of an adjuvant with or without the addition of tumour antigens. Despite the considerable activity in this area, there is still very little information on the effects of such therapy on general immunological reactivity as well as specific anti-tumour responses.

The subject of this report is a sequential analysis of some parameters of cellular immunological competence of patients with recurrent malignant melanoma and the effect of stage of disease and immunotherapy on these parameters.

### MATERIALS AND METHODS

*Patients.*—Thirty-six patients with malignant melanoma were referred to the Hamilton Clinic of the Ontario Cancer Treatment and Research Foundation for immunotherapy. Thirty-two of 35 had recurrent disease while 3 had primary lesions. The only criteria for exclusion were patients with other malig-

Address for reprints: Dr P. B. Dent, McMaster University Medical Center, 1400 Main St. West, Hamilton, Ontario L8S 4J9.

nancies (1 patient with chronic lymphocytic leukaemia) and patients over 70 years of age. Most patients were evaluated immunologically before surgery or immunotherapy if possible. Surgical excision of local or lymph nodal recurrences was performed wherever indicated. No other forms of therapy were used unless, for palliative purposes, radiation or chemotherapy was indicated in preterminal patients. Thirty-two normal adult controls were taken from a pool of laboratory and hospital personnel.

*Immunotherapy protocol.*—Bacillus Calmette-Guèrin (BCG) was obtained from Connaught Laboratories, Toronto, lyophilized in 40 mg vials. The equivalent of 5 mg of freshly reconstituted material was applied to the surface of one extremity. Six separate punctures were made through the topically applied vaccine with a multipronged Heaf Sterngun fitted with a disposable tip. Vaccination was applied weekly for 4 weeks, every 2 weeks for 8 weeks and then every 4 weeks. The viability of the organisms was confirmed by bacteriological culture. Three patients received one injection of autologous irradiated and neuraminidase treated tumour cells shortly after surgery. Regular physical examinations were carried out at the clinic and routine chest x-rays were performed every 2 months.

*Immunological studies.*—Total lymphocyte counts were performed monthly as part of the routine haematological assessment.

Purified protein derivative (PPD, Connaught Laboratories, 5TU) skin tests were performed before starting therapy and at monthly intervals thereafter. Candida (Hollister-Stier Laboratories, 1 : 100) and mumps (Eli Lilly and Co., 2 u) were administered before starting therapy and at 3-monthly intervals thereafter. Initial skin tests were read by one of us, but subsequently the patients were instructed in reading their own skin tests with the aid of a card template. To confirm the presence of anergy, contact sensitization with DNCB was performed (Catalona *et al.*, 1972).

Peripheral blood lymphocytes were cultured without separation using a modification of the method of Junge *et al.* (1970). Each culture consisted of 0·05 ml of heparinized whole blood in 1·95 ml of RPMI 1640 without added antibiotics or serum.

Mitogen responses were assessed by the addition of phytohaemagglutinin (PHA-P, Difco) in final concentrations previously determined to give (a) maximal and (b) threshold stimulation responses in normal subjects. These concentrations varied from bottle to bottle of PHA and had to be individually determined for each lot. A typical dose response curve based on titrations of one lot of PHA with 13 normal donors is shown in Fig. 1. The concentrations used for optimal and threshold stimulation would be 10 and 0·3 $\mu$g/ml respectively. Triplicate cultures were incubated at 37°C in 5% $CO_2$ for 67 h, at which time 2 $\mu$Ci of tritiated thymidine (specific activity 6·7 Ci/mmol) were added to each culture. After 5 h further incubation, the cultures were terminated by the addition of 450 $\mu$g of cold thymidine and were placed immediately at −20°C.

Incorporation of radioactive thymidine was determined by a previously described method (Dent, 1971), with the modification that the cell pellet containing intact erythrocytes was washed once with 3% acetic acid and once with normal saline before digestion with trichloracetic acid.

Proliferative responses to PPD *in vitro* were measured with the same technique, except that cultures were exposed to PPD (Connaught Laboratories-CT68) for 115 h before pulsing with tritiated thymidine. PPD was used at a final concentration of 10 and 1 $\mu$g/ml.

*Statistical analysis.*—Comparisons between patient groups and controls were made using the Student's unpaired $t$ test. To determine the effect of BCG on the parameters measured, the pre-BCG response was compared with the mean of the post-BCG responses using the paired $t$ test. A $P$ value of $<0·05$ was considered significant.

### RESULTS

### *Clinical staging*

The patients were divided into 4 groups. Group A (83 observations) included all patients without evidence of residual disease at the time of testing. The majority of these patients had had recurrences which were removed surgically. Group B (53 observations) included patients with local recurrences and/or regional node involvement. Group C (28 observations) included patients with

FIG. 1.—Dose response of PHA concentration and $^3$H-thymidine incorporation expressed as ct/min ($\pm 1$ s.e.).

metastatic disease beyond the regional lymph nodes. Group D (10 observations) was a retrospective categorization and included observations performed in the 6-week preterminal period before the death of the patient.

*Peripheral lymphocyte levels*

The normal total circulating lymphocyte level was 1906/mm$^3$. In Fig. 2 it can be seen that there is a progressive decrease in circulating lymphocyte count with advancing disease. The lymphocyte level of patients in Group C is 1346/mm$^3$ which is significantly lower than that of controls ($P < 0{\cdot}01$). There is a slight rise in preterminal patients (Group D) but this is not significantly different from the mean lymphocyte level of patients in Group C.

*Delayed hypersensitivity skin tests*

Twenty-one of the 35 patients were PPD skin test negative at the outset of BCG immunotherapy and 17/21 converted to positivity as a result of immunotherapy. Following institution of BCG therapy, 5 patients became skin test positive to mumps or Candida antigen after having previously been non-reactive. Six patients became non-reactive to Candida or mumps following BCG therapy.

FIG. 2.—Absolute peripheral lymphocyte counts in patients with different stages of disease. Each point represents the mean ($\pm 1$ s.e.) value for each group. The cross hatched area represents the mean ($\pm 1$ s.e.) of normal control values.

As determined by the size of the PPD skin test reaction, no differences could be detected among Groups A, B and C.

*Phytohaemagglutinin stimulation*

The *in vitro* lymphocyte blastogenic response to optimum stimulating concentrations of PHA are shown in Fig. 3. The

FIG. 3.—Lymphocyte stimulation with optimal stimulating concentrations of phytohaemagglutinin. Each point represents the mean ($\pm 1$ s.e.) value for each group. The cross hatched area represents the mean ($\pm 1$ s.e.) of normal control values.

mean ($\pm 1$ s.d.) response of normal subjects was $52{,}745 \pm 31{,}334$ ct/min. It can be seen that the mean response of melanoma patients was consistently lower than that of controls and that the decreased response was proportional to the extent of disease. However, no statistically significant differences were observed in any group of patients.

Using suboptimal or threshold concentrations of PHA, a more striking and significant depression of responses was observed (Fig. 4), which was clearly related to the extent of disease.

The levels of unstimulated thymidine incorporation are shown in Fig. 5. Significantly depressed levels were seen in Groups A, C and D, but not in Group B.

*PPD stimulation*

In our culture system we have determined (unpublished observations) that tuberculin sensitivity *in vivo* in normal subjects is almost always associated with a minimum *in vitro* PPD stimulated $^{3}$H-thymidine uptake of 2000 ct/min. The mean ($\pm 1$ s.d.) uptake of normal tuberculin positive controls in response to 10 $\mu$g/ml of PPD was $17{,}168 \pm 19{,}943$ ct/min. The analysis of *in vitro* PPD stimulation tests of patients who had positive *in vivo* skin tests is shown in Fig. 6. It can be seen that at 10 $\mu$g/ml of PPD, patients in Group B had significantly elevated levels of stimulation, while at concentrations of 1 $\mu$g/ml significantly increased stimulation was seen in Groups A, B and C. Most patients in Group D were anergic and too few results were obtained for meaningful analysis. Anergy was, however, associated with very low levels of $^{3}$H-thymidine uptake in response to PPD.

Fig. 4.—Lymphocyte stimulation with threshold stimulating concentrations of phytohaemagglutinin. Each point represents the mean ($\pm 1$ s.e.) value for each group. The cross hatched area represents the mean ($\pm 1$ s.e.) of normal control values.

### *Effect of BCG therapy*

The effect of BCG therapy on the immunological parameters studied are summarized in Fig. 7. The values for circulating lymphocytes, optimal and threshold PHA response and *in vitro* PPD stimulation before and after therapy (mean of several estimations) were compared using the paired *t* test. The PPD results of pre-therapy skin test negative and positive patients are examined separately.

The only significant change occurred in those patients who converted from PPD negative to PPD positive. It is of interest that these patients never achieved the same degree of hyperreactivity which was noted in the patients who were PPD positive before BCG therapy. There was a consistent trend toward decreased reactivity in all other parameters in the post-BCG state but these differences did not reach statistical significance.

### DISCUSSION

These studies demonstrate clearly that with the techniques used, patients with malignant melanoma can be shown to develop decreased cell mediated immune reactions with increasing tumour burdens. The level of sensitivity of most of the parameters is such that significantly impaired responses are detected only in

Fig. 5.—Unstimulated $^{3}$H-thymidine incorporation. Each point represents the mean ($\pm 1$ s.e.) value for each group. The cross hatched area represents the mean ($\pm 1$ s.e.) of normal control values.

FIG. 6.—Lymphocyte stimulation with 10 µg/ml (left) and 1 µg/ml (right) of purified protein derivative. Each point represents the mean (±1 s.e.) value for each group. The cross hatched area represents the mean (±1 s.e.) of normal control values.

FIG. 7.—Effect of BCG immunotherapy on absolute peripheral lymphocyte counts, phytohaemagglutinin stimulation and PPD stimulation. Patients who were PPD positive before beginning therapy are analysed separately from those who were PPD negative. PRE refers to pre-therapy values; POST refers to mean of values obtained while on therapy. Statistical analysis was performed bv the paired Students' *t* test comparing PRE- and POST values.

pre-terminal patients. Only when threshold concentrations of PHA are used can impaired responses in patients with less advanced disease be demonstrated. The importance of using suboptimal mitogen concentrations in demonstrating quantitative reductions in PHA reactivity has been reported previously (Oppenheim, Blaese and Waldman, 1970; Hosking, Fitzgerald and Simons, 1971; Faguet,

Balcerzak and LoBuglio, 1973; Finkel and Dent, 1973). The mechanism of impaired reactivity to threshold PHA concentrations is not clear but it has been suggested that the response to threshold concentrations of PHA is dependent on macrophages (Oppenheim, Leventhal and Hersh, 1968). It may be, therefore, that the impaired threshold response noted in these studies reflects an abnormality of macrophages rather than lymphocytes, a finding in keeping with the recent report of abnormal monocyte chemotaxis in patients with cancer (Boetcher and Leonard, 1974). Further studies are required to determine if defective monocyte chemotaxis and impaired threshold PHA responses are directly related.

Previous studies of cell mediated immunity have not revealed any consistent defects in patients with malignant melanoma. Ziegler *et al.* (1969) found no impairment of response to skin tests, DNCB sensitization or PHA response *in vitro*. Siegler *et al.* (1973), in a more intensive study of patients with more far advanced disease, found a decrease in delayed hypersensitivity skin test reactivity. *In vitro* stimulation tests with PHA failed to reveal any abnormalities. Catalona and Chretien (1973) reported that 69% of patients with melanoma had impaired DNCB reactivity; 25% of these were totally anergic. There was no difference in the frequency of impairment among patients with localized or metastatic disease. Catalona, Sample and Chretien (1973) found no impairment of PHA reactivity in 17 patients with melanoma. For unexplained reasons, if patients with localized disease were analysed separately, abnormally low responses were seen compared with patients with widespread disease. Finally, in contrast to the results of Catalona *et al.* (1973), Golub, O'Connell and Morton (1974) reported that patients with melanoma as a group have a significantly depressed response to mitogen stimulation with a much lower rate of anergy to DNCB sensitization (18%). Their findings resemble more closely those which we have presented in this report.

The effect of BCG therapy on immunological reactivity is a subject of great importance since, apart from survival or measurable disease regression, it is another parameter by which to gauge the possible effectiveness of this form of therapy. Bluming *et al.* (1972) have reported that BCG therapy by dermal scarification had a nonspecific potentiating effect on development of contact sensitivity to picryl chloride and on the recall of skin test reactivity to other skin test antigens which was not seen when lower doses of BCG were given by intradermal inoculation. No change in peripheral lymphocyte levels was noted. Chess *et al.* (1973) reported an increase in *in vitro* responses to recall antigens but no increase in PHA or mixed leucocyte reactivity. Gutterman *et al.* (1973) reported an increase in size of existing skin test reaction sites as well as some conversions from negative to positive responses to recall antigens but no effect on *in vitro* responses to PHA or specific antigens, or on circulating lymphocyte levels. Sokal, Aungst and Han (1973) also reported an increase in skin test reactivity to recall antigens in patients with various forms of lymphoma treated with BCG. Our studies confirm the above findings relative to mitogen responses and lymphocyte levels. We did not carefully examine the response to recall antigens although 4 patients did show positive Candida or mumps responses following BCG therapy after having been skin test negative to these antigens before BCG administration.

At the time of writing, 15 of the 35 patients have died. Analysis of the data to determine if those patients who have died had initially lower responses failed to reveal any significant differences when compared with the initial values for those patients who are still alive. Impaired responses were more directly related to the extent of disease. At the frequency with which the tests were performed, we were unable to attach any predictive value to falling immune competence as such changes almost always were associated with clinical evidence of progression.

Finally, while we have shown that the extent of disease does correlate with a number of parameters of host responses, we do not feel that the information obtained is of any assistance in the management of the patient with cancer. As yet, there is little evidence that any form of immunotherapy produces a quantifiable effect on general immune competence that correlates with patient survival. While it is of interest, though not universally true (Chakravorty *et al.*, 1973; Greene, Schimpff and Wernik, 1974), that intact immune reactivity correlates with good prognosis (Harris and Copeland, 1974), this knowledge does little to help the clinician in making management decisions about individual patients. Until we are able regularly and predictably to influence immunocompetence of cancer patients, the routine and widespread performance of tests of nonspecific immunocompetence should be discouraged.

Supported in part by a grant from the Ontario Cancer Treatment and Research Foundation. The authors wish to thank Mrs B. Roberge and Miss Karen Winicki for their technical skill and Mrs Grace Bridle and Mrs Joyce Gillan for secretarial support. We are particularly grateful for the diligent and enthusiastic support of the nursing staff of the Ontario Cancer Foundation, Hamilton Clinic.

### REFERENCES

BLUMING, A. Z., VOGEL, C. L., ZIEGLER, J. L., MODY, N. & KAMYA, G. (1972) Immunological Effects of BCG in Malignant Melanoma: Two Modes of Administration Compared. *Ann. intern. Med.*, **76**, 405.

BOETCHER, D. A. & LEONARD, E. J. (1974) Abnormal Monocyte Chemotactic Response in Cancer Patients. *J. natn. Cancer Inst.*, **52**, 1091.

CATALONA, W. J., TAYLOR, P. T., RABSON, A. S. & CHRETIEN, P. B. (1972) A Method of Dinitrochlorobenzene Contact Sensitization Pathologic Study. *New Engl. J. Med.*, **2£6**, 399.

CATALONA, W. J. & CHRETIEN, P. B. (1973) Abnormalities of Quantitative Dinitrochlorobenzene Sensitization in Cancer Patients: Correlation with Tumor Stage and Histology. *Cancer, N.Y.*, **31**, 353.

CATALONA, W. J., SAMPLE, W. F. & CHRETIEN, P. B. (1973) Lymphocyte Reactivity in Cancer Patients: Correlation with Tumor Histology and Clinical stage. *Cancer, N.Y.*, **31**, 65.

CHAKRAVORTY, R. C., CURUTCHET, H. P., COPPOLLA, F. S., PARK, C. M., BLAYLOCK, W. K. & LAWRENCE, W. (1973) The Delayed Hypersensitivity Reaction in the Cancer Patient: Observations on Sensitization by DNCB. *Surgery, St Louis*, **73**, 730.

CHESS, L., BOCK, G. N., UNGARO, P. C., BUCHHOLZ, D. H. & MARDINEY, M. R. (1973) Immunologic Effects of BCG in Patients with Malignant Melanoma: Specific Evidence for Stimulation of the " Secondary " Immune Response. *J. natn. Cancer Inst.*, **51**, 57.

DENT, P. B. (1971) Inhibition by Phytohemagglutinin of DNA Synthesis in Cultured Mouse Lymphomas. *J. natn. Cancer Inst.*, **46**, 763.

EILBER, F. R. & MORTON, D. L. (1970) Impaired Immunologic Reactivity and Recurrence following Cancer Surgery. *Surgery, St Louis*, **25**, 362.

FAGUET, G. B., BALCERZAK, S. P. & LOBUGLIO, A. F. (1973) Lymphocyte Sensitivity and Cellular Immunity in Neoplasia. *Biomedicine*, **19**, 43.

FINKEL, A. & DENT, P. B. (1973) Abnormalities in Lymphocyte Proliferation in Classical and Atypical Measles Infection. *Cell. Immunol.*, **6**, 41.

GOLUB, S. H., O'CONNELL, T. X. & MORTON, D. L. (1974) Correlation of *in vivo* and *in vitro* Assays of Immunocompetence in Cancer Patients. *Cancer Res.*, **34**, 1833.

GREENE, W. H., SCHIMPFF, S. C. & WERNIK, P. H. (1974) Cell-mediated Immunity in Acute Nonlymphocytic Leukemia: Relationship to Host Factors, Therapy and Prognosis. *Blood*, **43**, 1.

GUTTERMAN, J., MAVLIGIT, G., MCBRIDE, C., FREI, E. & HERSH, E. M. (1973) BCG Stimulation of Immune Responsiveness in Patients with Malignant Melanoma. *Cancer, N.Y.*, **32**, 321.

HARRIS, J. & COPELAND, D. (1974) Impaired Immunoresponsiveness in Tumor Patients. *Ann. N. Y. Acad. Sci.*, **230**, 56.

HOSKING, C. S., FITZGERALD, M. G. & SIMONS, M. J. (1971) Quantified Deficiency of Lymphocyte Response to Phytohemagglutinin in Immune Deficiency Diseases. *Clin. & exp. Immunol.*, **9**, 467.

JUNGE, U., HOEKSTRA, J., WOLFE, L. & DEINHARDT, F. (1970) Microtechnique for Quantitative Evaluation of *in vitro* Lymphocyte Transformation. *Clin. & exp. Immunol.*, **7**, 431.

OPPENHEIM, J. J., LEVENTHAL, B. G. & HERSH, E. M. (1968) The Transformation of Column-purified Lymphocytes with Nonspecific and Specific Antigenic Stimuli. *J. Immun.*, **101**, 262.

OPPENHEIM, J. J., BLAESE, R. M. & WALDMANN, T. A. (1970) Defective Lymphocyte Transformation and Delayed Hypersentivity in Wiskott-Aldrich Syndrome. *J. Immun.*, **104**, 835.

SIEGLER, H. F., SHINGLETON, W. W., METZGAR, R. S., BUCKLEY, C. E. & BERGOC, P. M. (1973) Immunotherapy in Patients with Melanoma. *Ann. Surg.*, **178**, 352.

SOKAL, J. E., AUNGST, C. W. & HAN, T. (1973) Effect of BCG on Delayed Hypersensitivity Responses of Patients with Neoplastic Disease. *Int. J. Cancer*, **12**, 242.

ZIEGLER, J. L., LEWIS, M. G., LUYOMBYA, J. M. S. & KIRYABWIRE, J. W. M. (1969) Immunologic Studies in Patients with Malignant Melanoma in Uganda. *Br. J. Cancer*, **23**, 729.